# Structural, Magnetic and THz Emission Properties of Ultrathin Fe/L1_0_-FePt/Pt Heterostructures

**DOI:** 10.3390/nano15141099

**Published:** 2025-07-16

**Authors:** Claudiu Locovei, Garik Torosyan, Evangelos Th. Papaioannou, Alina D. Crisan, Rene Beigang, Ovidiu Crisan

**Affiliations:** 1 National Institute of Materials Physics, Atomistilor 405A, 077125 Magurele, Romania; claudiu.locovei@infim.ro (C.L.);; 2 Department of Electrical and Computer Engineering and Research Center OPTIMAS, Rheinland-Pfälzische Technische Universität Kaiserslautern-Landau, 67663 Kaiserslautern, Germany; garik.torosyan@rptu.de; 3 Department of Physics, Aristotle University of Thessaloniki, 54124 Thessaloniki, Greece; 4 Department of Physics, Rheinland-Pfälzische Technische Universität Kaiserslautern-Landau, 67663 Kaiserslautern, Germany; rene.beigang@icloud.com

**Keywords:** THz emission, magnetic heterostructures, L1_0_-FePt

## Abstract

Recent achievements in ultrafast spin physics have enabled the use of heterostructures composed of ferromagnetic (FM)/non-magnetic (NM) thin layers for terahertz (THz) generation. The mechanism of THz emission from FM/NM multilayers has been typically ascribed to the inverse spin Hall effect (ISHE). In this work, we probe the mechanism of the ISHE by inserting a second ferromagnetic layer in the form of an alloy between the FM/NM system. In particular, by utilizing the co-sputtering technique, we fabricate Fe/L1_0_-FePt/Pt ultra-thin heterostructures. We successfully grow the tetragonal phase of FePt (L1_0_-phase) as revealed by X-ray diffraction and reflection techniques. We show the strong magnetic coupling between Fe and L1_0_-FePt using magneto-optical and Superconducting Quantum Interference Device (SQUID) magnetometry. Subsequently, by utilizing THz time domain spectroscopy technique, we record the THz emission and thus we the reveal the efficiency of spin-to-charge conversion in Fe/L1_0_-FePt/Pt. We establish that Fe/L1_0_-FePt/Pt configuration is significantly superior to the Fe/Pt bilayer structure, regarding THz emission amplitude. The unique trilayer structure opens new perspectives in terms of material choices for the future spintronic THz sources.

## 1. Introduction

### 1.1. State of the Art

Spin dynamics at ultrafast timescales together with spin-to-charge conversion mechanisms such as spin Hall effect (SHE) and the inverse spin Hall effect (ISHE) are at the center of research nowadays in the field of spintronics since they have tremendous potential to impact technologically and to interconnect spintronics with optics and photonics, phononics and electronics. One particular example is the case of terahertz (THz) spintronics. The latter deals with nanostructures composed of ferromagnetic and non-magnetic metallic bilayers, trilayers and multilayers, so-called spintronic THz emitters (STEs) [1]. The non-magnetic (NM) layer is usually a heavy metal with high spin-orbit coupling and consequently a large enough spin Hall angle [1,2,3,4,5,6,7]. When a STE heterostructure is illuminated by femtosecond (fs) laser pulses, an ultrafast spin-polarized current in the FM layer is excited, which is subsequently injected into the NM layer. There, it can be converted into a transverse charge current via the relativistic ISHE. This ultrafast transient charge current then generates THz radiation [1,2,3,4,5,6,7].

The properties of the THz emission that STEs provide hold the promise for applications in the next generation of THz technologies. STEs offer high THz field strengths [8,9] spatiotemporal modulation of the THz beam [10,11], easy implementation even on flexible substrates and on fiber tips [12,13], and an exceptionally broad spectrum extending up to 30 THz [2,14].

Despite the achievements of STEs, the optical-to-terahertz conversion efficiency is still considerably lower than that of traditional THz sources based on semiconductors, with the exception of non-linear crystals [8]. Typical values of efficiency are in the order of 10^−5^–10^−6^ with respect to the pumping energy [15,16]. The challenge to increase the efficiency has been addressed by materials [1,2,7,17], interfaces [18,19,20] and different optical environments that maximize the electromagnetic efficiency independent of the ultrafast spin dynamics [8,9].

In this work, we address the efficiency of THz emission by focusing on the intrinsic strength of the inverse spin Hall effect (that governs the effect of THz emission) in heterostructures in the form of FM1/FM2/Pt, where FM1 denotes ferromagnetic layer 1 and FM2-ferromagnetic layer 2. In particular, here, FM1 is Fe and FM2 is FePt alloy, while NM is represented by Pt.. Contrary to previous studies on alloyed interfaces [19], the FM2 alloy layer is not induced by thermal annealing but it is directly deposited on Fe using the co-sputtering technique. In this way, we can precisely control the thickness of the FM2 layer and explore the role of the alloy in the ISHE and the THz emission. We show that the co-deposition on Fe buffer layer leads to the creation of an L1_0_-FePt alloy phase. We modify the thickness of the Fe layer to study the influence on the spin current that is pumped away from the FM1 layer. The Fe/L1_0_-FePt/Pt obtains unique magnetic properties since Fe/L1_0_-FePt are magnetically coupled via exchange spring effect. We further test the structures in terms of their ability to perform spin-to-charge conversion. We quantify this conversion with THz spectroscopic measurements. We report that Fe/L1_0_-FePt/Pt is very efficient spin current converter and THz emitter. The unique trilayer structure opens new perspectives in terms of material choices for the next generation of spintronic THz emitters.

### 1.2. Principle of Method

**Figure 1:**
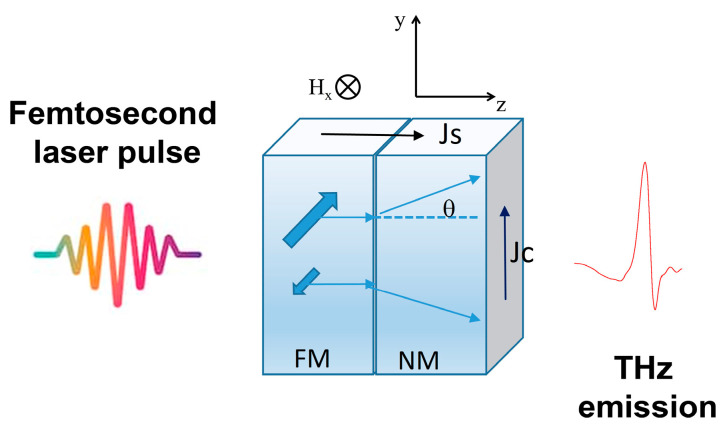
Schematic of the THz emission by optically exciting a FM/NM structure with femtosecond laser pulse based on the inverse spin Hall effect mechanism. J_S_ is the excited spin current, J_C_ the transient charge current, θ the spin Hall angle.

The physical origin of the THz emission after fs-laser excitation in magnetic heterostructures has been attributed to different mechanisms. The principle of THz emission in spintronic structures based on the inverse spin Hall effect (ISHE) is shown in Figure 1. Upon irradiation with a femtosecond laser of the ferromagnetic (FM) layer, electrons in the FM-layer are excited from quasi-localized states (*d*-like states) beneath the Fermi level to more mobile states (*sp*-like states) above the Fermi level. The properties of the excited electrons such as the lifetime and velocity depend on the energy and the spin type (majority or minority). Majority spins are more mobile than minority spins therefore more majority carriers are available near the Fermi level. The imbalance in the number of majority and minority spins gives rise to an ultrafast spin polarized current, j_S_, that propagates towards the non-magnetic metallic layer through a superdiffusive process [21,22,23]. The source of spin current has been attributed to several different physical mechanisms [24] with the superdiffusive model being the most accepted one [24]. This longitudinal spin polarized current j_S_ enters the non-magnetic (NM) layer that has large spin-orbit coupling. This causes the spin-up and spin-down electrons to be deflected in opposite directions by an angle Θ. Henceforth, an inverse spin Hall effect voltage is produced, associated with the conversion of the spin current density j_S_ into ultrafast charge current density j_c_ = Θ j_s_. The ultrafast transient electric field gives rise to short terahertz pulses, of energy $E_{THz}=\frac{\partial \int j_{C}}{\partial t}$, that can be detected using electro-optical sampling (EOS) or photoconductive antenna (PCA) methods using time-domain terahertz spectrometry setups (TDS-THz) [1,14].

However, the ISHE mechanism is not the only one that can lead to the emission of THz radiation. Presently, new spin-to-charge conversion mechanisms have been proposed as sources of THz radiation. One example is the THz emission from Rashba type interfaces FM/NM1/NM2 [25,26], where in a similar experimental procedure, the fs-laser pulse induces a femtosecond spin current pulse in the FM layer that then drives terahertz transients at a Rashba interface between two nonmagnetic materials. Furthermore, an alternative mechanism for generating THz emission from an ultrathin FM layer via the anomalous Hall effect was recently demonstrated [27,28,29]. The process involves a single FM layer and the generation of backflow superdiffusive currents at the dielectric/FM/dielectric interfaces and subsequent conversion of the charge current to transverse current due to the anomalous Hall effect. In addition, very recently, the role not only of the spin momentum but also of the orbital momentum has been shown to lead to THz generation [30,31]. The variety of mechanisms in spintronic emitter that lead to THz radiation also include optical rectification effects as shown in Heusler alloys/NM [32,33] or spin-to-charge conversion in topological interfaces in Bi_2_Se_3_/Co heterostructures [34] or even more, by surface optical rectification in indium tin oxide thin films [35].

## 2. Materials and Methods

### 2.1. Fabrication

Direct current magnetron sputtering (DC-MS) is a versatile synthesis technique suitable for deposition of high-quality thin films, multilayers and other nanosystems with thicknesses ranging from few monolayers up to microns. The sputtering facility used for the synthesis of the films is a S16 Physical Vapor Deposition PVD UHV system with cylindrical upper opening chamber from Intercovamex, Querétaro, Mexico, furnished with four radially mounted magnetrons, driven by either DC or RF power source. The targets are horizontally mounted on magnetron’s active surface. For achieving the desired deposition, we used elemental targets such as Pt (purity 99.99%) and Fe (purity 99.95%). For this study, the substrates were used as received from MaTecK Material-Technologie & Kristalle GmbH, Julich, Germany. The substrate consisted of MgO <001> single crystal with dimensions 10 mm × 10 mm and for each sample the sequence FM1/FM2/NM were grown. Substrate was mounted on the sample holder disposed inside the chamber, in the upper part, at approximately 12 cm from each radially disposed targets. Initial vacuum in the chamber was lower than 2 × 10^−8^ torr. Fe-Pt films were then sputtered in high purity Ar gas at a working pressure of 5.8 to 7 × 10^−3^ torr. The pressure we used was previously calibrated to ensure optimal rate of evaporation. The growth rates, between R = 0.25 Å/s and R = 1 Å/s, were controlled by a quartz crystal during the deposition procedure in which the incident sputtered beam was oriented perpendicular to the MgO substrate. Depending on the desired composition, electrical powers between 25 W and 40 W in the case of Pt and between 50 W and 90 W in case of Fe target were applied on the magnetrons. The exposure times were varied depending on the desired thickness of the layer. In a typical sputtering sequence, first step was with Fe target kept on while Pt target was kept off (non-emitting), then in the second step Fe and Pt targets were kept on, meaning emitting sputtered atoms from the target surface to the heated substrate, for achieving the desired thickness, while in the final step, Fe target was kept off and Fe target was emitting, in order to achieve the deposition on top of the device of a uniform Pt layer. Samples were then retrieved from the deposition chamber and stored under primary vacuum to avoid oxidation during slow cooling after deposition.

### 2.2. Investigation Techniques

**Figure 2:**
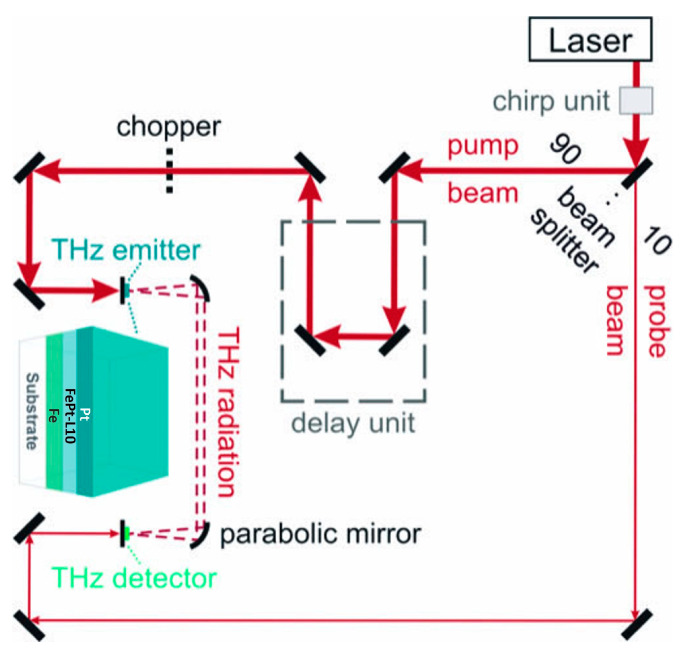
Schematic of the THz time-domain spectroscopy (THz-TDS) measurement setup. The spintronic bilayer used as the source of the THz radiation is shown in magnification. The probe beam was used to excite a photoconductive antenna (PCA) acting as THz detector.

Structural characterization was extensively performed using X-ray diffraction (XRD) as well as X-ray reflectivity (XRR). The XRD analysis was carried out using a Rigaku SmartLab diffractometer (from Rigaku Europe SE, Neu-Isenburg, Germany) with Cu Kα1 radiation. Measurements were performed employing a Ge(400) 2-bounce channel-cut monochromator mounted into the incident X-ray beam path with the wavelength $\lambda_{K_{\alpha 1}}^{Cu}=1.5406\;Å$. The geometry of the measurements was 2θ−ω and measurements were taken after the sample alignment on the (002) plane of MgO single crystal substrate. Magnetic characterization has been performed using magneto optic Kerr effect (MOKE) magnetometry as well as with a Superconducting Quantum Interference Device (SQUID). The MOKE measurements have been done at ambient temperature using a longitudinal magneto-optic Kerr effect magnetometer, with zero remanence and an incident p-polarized He-Ne laser light with λ = 633 nm. The incident light made a 45° angle with the sample plane, being linearly polarized perpendicular to the incidence plane, through a polarizer. The MOKE effect describes the electromagnetic wave interaction with the magnetic material, which corresponds to the rotation or changing the linear polarized light intensity by reflection on magnetic surfaces placed in a magnetic field and is proportional with the specific magnetization of the film. Magnetic properties were derived by measuring major hysteresis loops via SQUID measurements under an applied magnetic field of up to 2 T applied parallel and perpendicular to the sample plane at room temperature. The THz experiments were performed using a standard THz time-domain spectroscopy (THz-TDS) system, where the trilayers were used as THz emitters [8]. The scheme of the THz-TDS experimental setup is presented in Figure 2.

The laser beam is split into a pump and probe beam usually by a 90:10 beam-splitter. The stronger part is led through a mechanical computer-controlled delay line to pump the THz emitter. The femtosecond (fs) Ti:Sa laser produces optical pulses of 22 fs length at a wavelength of 800 nm with a repetition rate of 80 MHz and a typical average output power of 1000 mW. The probe beam is used to excite a photoconductive antenna (PCA) that acts as a THz detector. The spintronic emitter is magnetized by a permanent external magnetic field of maximum value of 30 mT. The measurements were performed at room temperature at air conditions. The recorded voltage is proportional to the momentary electric field amplitude of the THz wave. The alignment of the optical beam path and of the components is not changed during exchange of the different spintronic emitter samples rendering the relative measurements comparable.

## 3. Results and Discussion

### 3.1. Structure and Schematics of Sputtering of THz Devices

**Figure 3:**
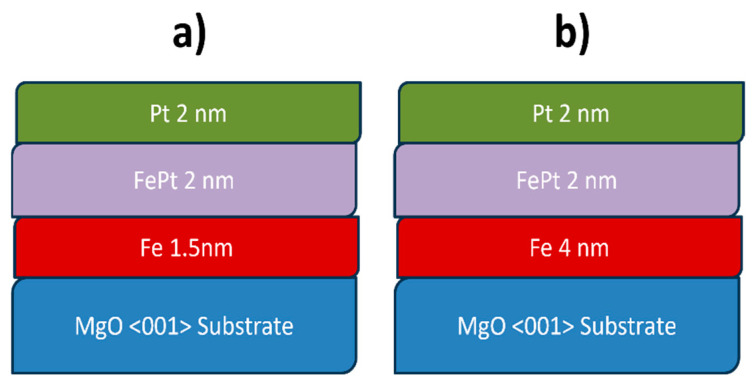
Cross-section sequence of deposition of the two FM1/FM2/NM trilayer devices: (**a**) S1 and (**b**) S2.

Two heterostructure samples in a FM1/FM2/NM sequence, or more precisely Fe/FePt/Pt, named S1 and S2, were prepared on MgO <001> substrates by DC-MS with the Intercovamex S16 system. Both deposited samples in the order Fe/FePt/Pt have the sequence of layers as seen in Figure 3. The iron thin film was prepared from a 2 inch target of Fe (99.95% purity), and the platinum layer was synthesized from a Pt (99.99% purity) target with the same diameter. By co-deposition from Fe and Pt targets, the FePt alloy was obtained and the composition was controlled via applied power on each target (see Table 1). Sample stage was rotating in all depositions to ensure good uniformity of thin films. The alternating sequence of deposition of the two FM1/FM2/NM trilayer devices, S1 and S2, as well as their thicknesses, are illustrated in Figure 3.

The technological parameters of the deposition of FM1/FM2/NM trilayer devices, during the sputtering experiments, namely power applied to each target, temperature of the substrate, nominal thickness as well as the working pressure during deposition, are schematized in Table 1, for both S1 and S2 samples.

**Figure 4:**
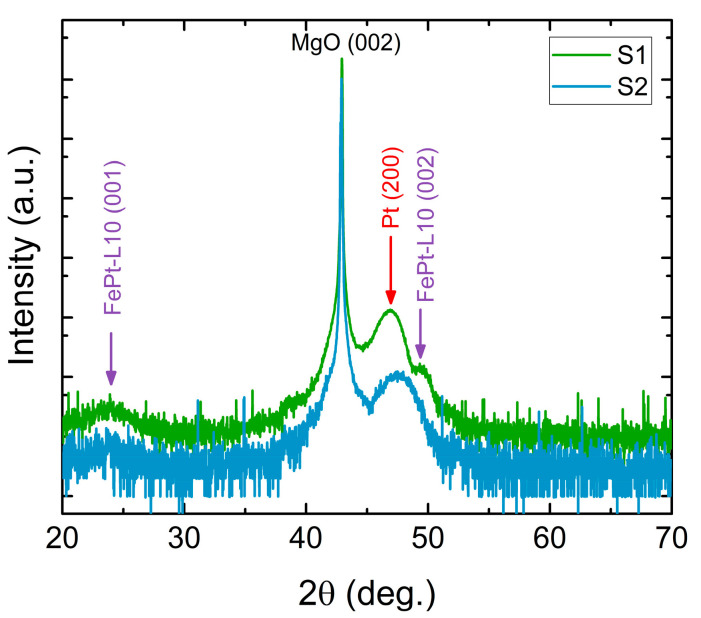
X-ray diffraction measurements of samples S1 and S2. The geometry of the measurements was 2θ−ω and the sample is aligned along the (002) plane of MgO single crystal substrate.

The structural investigation was performed through X-ray diffraction (XRD) in ambient conditions. The two diffractograms obtained for S1 and S2 are presented in Figure 4. Here one can observe the occurrence of the face centered cubic *fcc* Pt and tetragonal L1_0_-FePt phases as witnessed by the Bragg lines illustrated in the figure: the (002) reflection of the tetragonal L1_0_-FePt as well as the (200) reflection of the *fcc* Pt. It can be seen that of all the possible reflections, only the (00l) planes are observed for L1_0_-FePt in the diffractogram, a fact that indicates the lack of polycrystalline character and the very good quality of the deposited films. As such, an epitaxial growth of Pt and FePt thin films on the MgO <001> is inferred. The XRD data of sample S2 show a broad peak centered between the peaks of Pt (200) and L1_0_-FePt (002) which can envelope the scattering intensity from Pt (200) and L1_0_-FePt (002). The overlapping of these two diffraction lines is due to the large linewidth of the Bragg peaks associated with the nanometric size of the trilayer. This reveals a lower structural order than in the sample S2. Bragg lines attributable to the body-centered-cubic *bcc* Fe is not detected in either sample, regardless of the Fe thickness of the two samples (1.5 and 4 nm, respectively).

**Figure 5:**
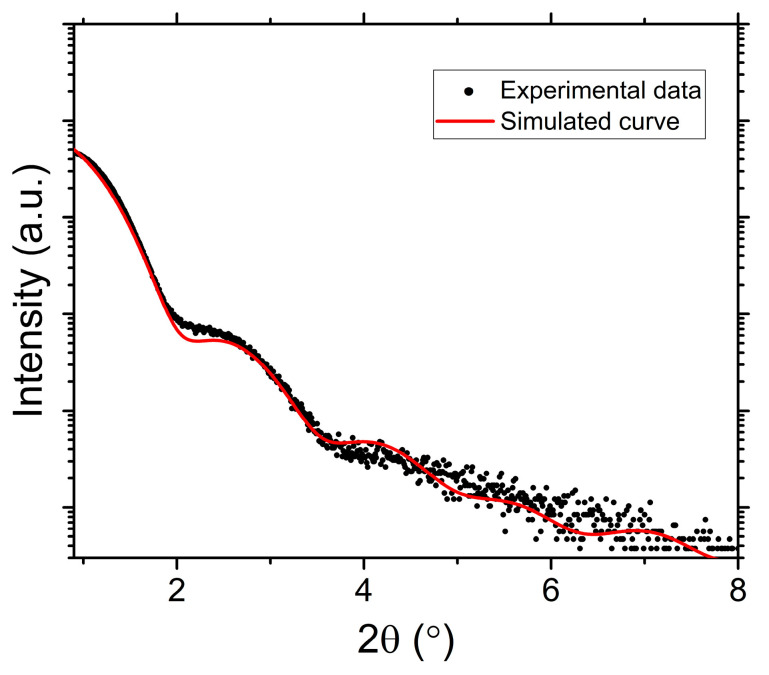
X-ray reflectivity curve recorded for sample S1. Measurements were taken at room temperature.

Furthermore, sample S1 was analyzed by X-ray reflectometry (XRR), as shown in Figure 5. Parameters extracted from simulated curve of XRR data confirms the 3-layer character of the device. The obtained thickness/roughness from XRR for each layer are the following: Fe 0.8/0.15 nm, FePt 3.7/0.13 nm, and Pt 1.1/0.16 nm. Noteworthy, a chemical diffusion is observed from both interfaces of FePt layer with the bottom Fe layer and the top Pt layer, resulting in a gradient of density values across the device thickness. As such, the bottom density is calculated to be 12.5 g/cm^3^ while the top density has the value of 19.3 g/cm^3^. By comparing the XRD patterns in Figure 4 one can successfully argue that the Fe layer (0.8 nm) acts as a buffer layer and furthermore, it improves the degree of structural ordering in both tetragonal FePt-L1_0_ and *fcc* Pt.

### 3.2. Magnetic Characterization of THz Devices

**Figure 6:**
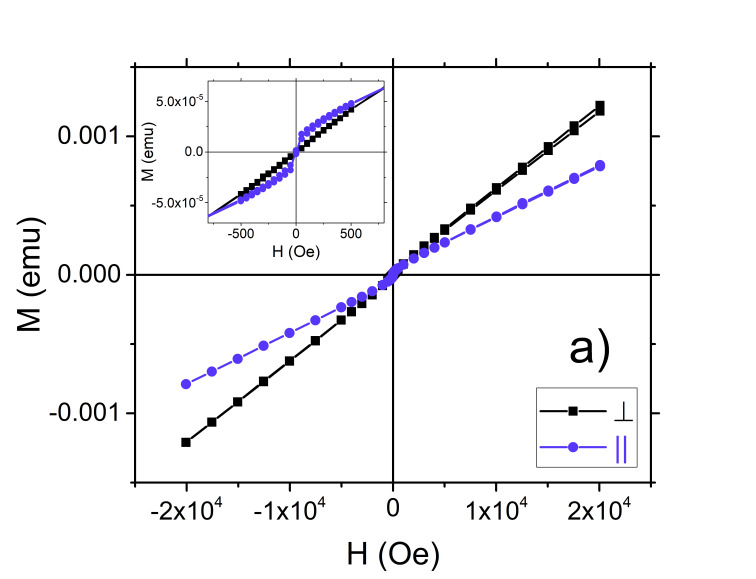

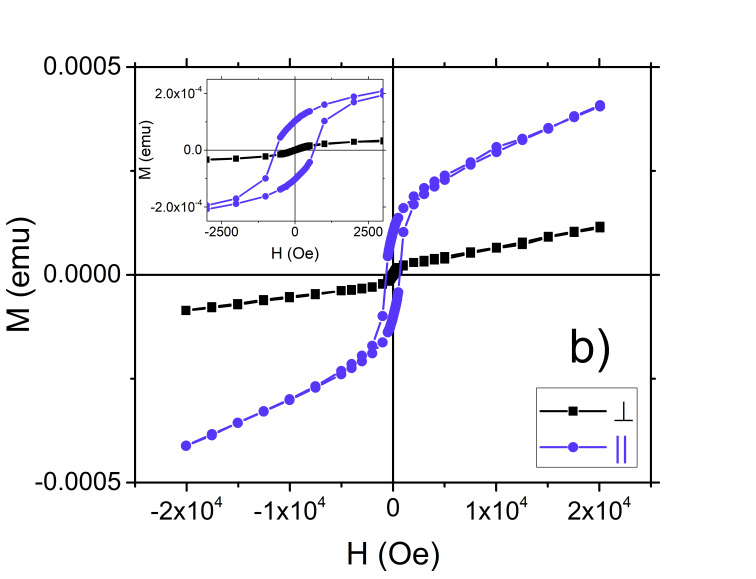
Room temperature hysteresis loops collected from SQUID for (**a**) S1 and (**b**) S2 in perpendicular and parallel orientation of the external magnetic field with respect to substrate surface. In inset is shown a close-up view of hysteresis loops around the origin of the graph.

The magnetic properties were studied by SQUID magnetometry at room temperature, by measuring the initial magnetization as a function of applied field as well as hysteresis loops. Measurements were performed with the applied field oriented both parallel as well as perpendicular to the sample plane (see Figure 6). In it observed that both samples S1 and S2 have a typically ferromagnetic behavior, showing an in-plane easy axis of magnetization. This can be attributed to the competition between the strong macroscopic perpendicular anisotropy of the FePt-L1_0_ phase and the shape anisotropy of the thin films, which favors in-plane magnetization due to demagnetization effects. The exchange interaction across the Fe/FePt interface produces an effective coupling between the two layers through an exchange spring mechanism. This translates into the fact that the magnetization of the soft magnetic Fe layer rotates reversibly under externally applied magnetic fields, in the same time remaining coupled via exchange interaction to the magnetization direction of the hard magnetic FePt layer.

Taking into account that in the sample S1, a significant amount of iron and platinum atoms diffuse into FePt layer through both Fe/FePt and FePt/Pt interfaces, a compositional gradient across the heterostructure thickness is obtained, as was previously revealed from XRR results. Therefore, after the diffusion through interfaces, the actual Fe layer is thicker than the initial nominal value of 4 nm for the S2 sample. This leads subsequently to a more pronounced in-plane magnetic anisotropy for the S2 sample.

In Figure 6b the coercive field for the in-plane hysteresis loop has a value of 650 Oe. The rounded loop shape further implies non-uniform magnetization reversal, likely involving spin coherent rotation as in exchange-spring systems. In perpendicular geometry the magnetization is gradually rotated by external magnetic field without any coercive field. Also, the shape of the hysteresis loops in Figure 6 illustrates a potential two-phase magnetic structure. One phase saturates in lower field while the other does not reach the saturation in the maximum applied magnetic field of 2T. The hysteresis loop acquired in parallel orientation from Figure 6a presents a soft-magnetic character, coexisting with a non-saturated magnetic phase, of a potential antiferromagnetic character.

The in-plane magnetic anisotropy of the two FM1/FM2/NM trilayer devices deposited by DC-magnetron sputtering was studied using vectorial Magneto-Optical Kerr Effect (MOKE), by measuring hysteresis loops as function of azimuthal angles between the applied magnetic field and a fixed reference direction within the film plane (in-plane axis).

**Figure 7:**
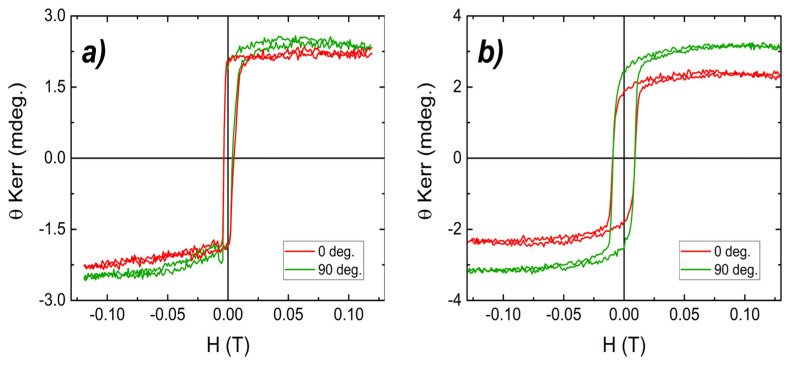
MOKE hysteresis loops taken at room temperature an azimuthal angle of 0° and 90° of samples (**a**) S1 and (**b**) S2.

The hysteresis loops acquired using MOKE depicted in Figure 7 show no preferential direction of magnetic anisotropy, for instance in-plane magnetic anisotropy, for none of the investigated samples, S1 and S2. The initial magnetization saturates quite fast, at very low applied magnetic fields. Values of applied field where the magnetization saturates are demonstrated to be: around 35 Oe in sample S1 and 220 Oe in sample S2, respectively. Moreover, a quite pronounced coercivity is recorded for the sample S2. Here, the measured value for the coercive field is around 90 Oe. In the case of hysteresis loops recorded from SQUID, a larger value of coercive field for the sample S2 is obtained, this being probably due to the wider range of the applied field. The MOKE hysteresis curves were measured in ±1200 Oe compared with the ones from SQUID collected in ±20,000 Oe. Nonetheless, the saturation is reached from a gradual rotation of magnetization in sample S2 and a sharp transition from positive to negative values of the Kerr angle is observed in S1 trilayer device, as presented in the SQUID hysteresis loops.

### 3.3. Evidencing Spin to Current Conversion Through THz Emission from the Trilayers

**Figure 8:**
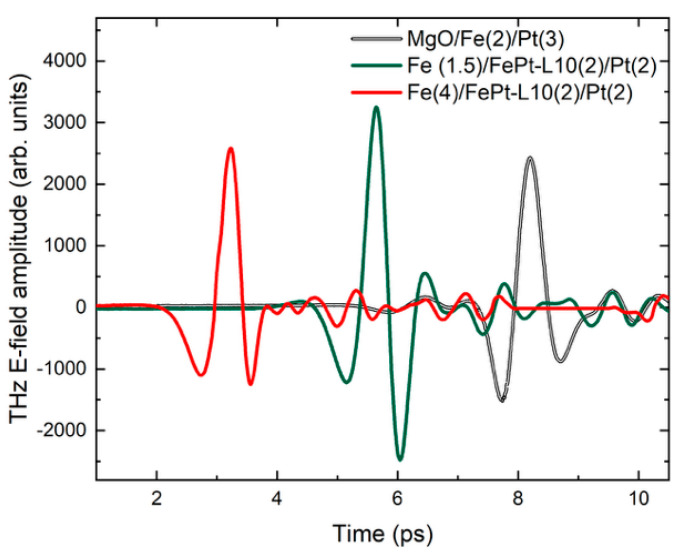
THz E-field amplitude emission from the two Fe/L1_0_-FePt/Pt samples (S1 and S2) as well as the emission from a reference MgO/Fe(2)/Pt(3) bilayer. The pulses are shifted in the time axis for clarity.

The spin-to-charge conversion (SCC) in Fe/L1_0_-FePt/Pt heterostructures on ultrafast timescales enabled by the inverse spin Hall effect (ISHE) was studied via THz time domain spectroscopy technique [1]. The measurements have been performed by first illuminating the metal side and then detecting the signal from the substrate side. The emission pulses of the S1 and S2 samples are presented in Figure 8. Here, we compare Fe (1.5 nm)/L1_0_-FePt (2 nm)/Pt (2 nm) and Fe (4 nm)/L1_0_-FePt (2 nm)/Pt (2 nm) with a reference Fe (2 nm)/Pt (3 nm) bilayer also grown on MgO. The latter exhibits the maximum signal in Fe/Pt bilayers with respect to the Fe and Pt thicknesses [36]. The geometry of the experiment implies that laser pulses illuminate firstly the metal (Pt) side. The measurements were performed at RT under air conditions with air humidity in the range of 25%.

**Figure 9:**
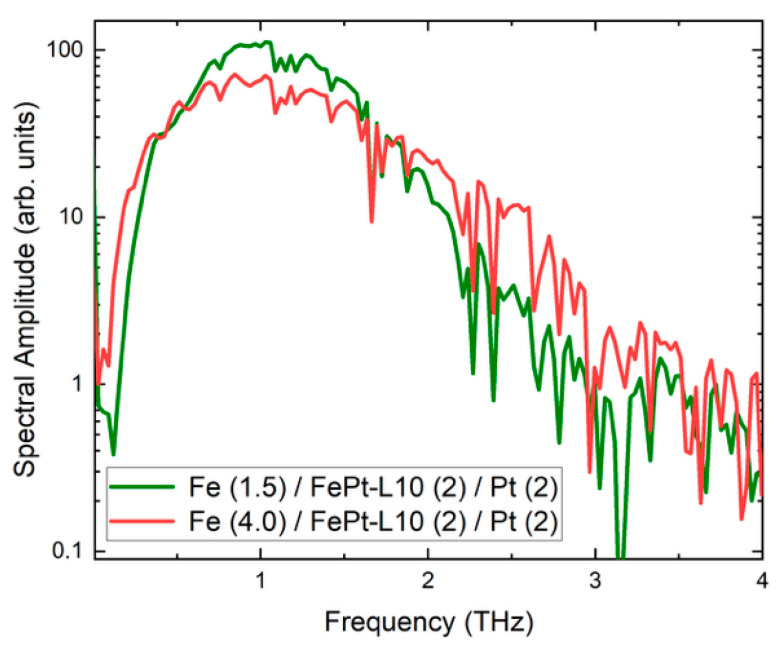
Frequency spectral amplitude of the two Fe/L1_0_-FePt/Pt samples (S1 and S2) samples, derived by fast Fourier transformation from the THz E-field emission spectra of Figure 8.

A very interesting result is that the emission amplitude (peak-to-peak) of sample S1, or Fe (1.5 nm) / L1_0_-FePt (2 nm)/Pt (2 nm) surpasses the signal of the reference Fe(2)/Pt(3) bilayer a factor of 1.44, a result which represents one of the highlights of this paper. The presence of the L1_0_ phase at the Fe/Pt interface enhances the spin-to-charge conversion as the enhanced THz signal reveals. The effect is sensitive to the thicknesses of the Fe layers and the subsequent magnetic coupling to the L1_0_-phase. The S2 sample, or Fe (4 nm)/L1_0_-FePt (2 nm)/Pt (2 nm), performs similar to the bilayer Fe(2)/Pt(3) reference sample. The reduced emission compared to the sample S1 is also attributable to the higher Fe thickness, as it has been argued [36] that thicker layers can reduce the THz emission. In Figure 9, the corresponding frequency spectra of the time-domain THz signals is presented. The spectral width is around 3 THz while the signal-to-noise ratio (SNR) was 200. The bandwidth is limited to the range provided by the GaAs photoconductive antenna while the humidity level of about 25% has contributed to the observed SNR.

The large difference in the amplitude of the THz radiation shows the important role of the presence of the L1_0_ FePt phase in transmission of the spin current. Our results confirm the tendency for THz enhancement in the presence of the L1_0_ phase, first observed in [16], but for much thicker Fe and Pt layers. In the present paper, we managed to prove this enhancement of the THz emission in much thinner layers. Furthermore, we prove that the co-sputtering technique is an easier way to achieve such structures and to control the thickness of the L1_0_ phase that is not possible with the thermal annealing used previously [16]. The enhancement seems to be very sensitive to the crystal quality of the L1_0_ phase. The crystallinity as proven by the X-ray diffraction patterns in Figure 4 is higher in sample S1 which shows stronger and clearer L1_0_ phase peaks for the Fe (1.5 nm)/L1_0_-FePt (2nm)/Pt (2nm) case. Furthermore, the magnetic coupling between Fe/L1_0_-FePt can also play a significant role to the THz emission efficiency. It seems that the magnetic hardening of the Fe (4 nm)/L1_0_-FePt (2nm)/Pt (2nm) that is attributed to the stronger coupling between Fe(4) and L1_0_-FePt (2) does not facilitate the spin current transparency of the L1_0_-FePt/Pt interface.

## 4. Conclusions

In the present work, we demonstrate strongly enhanced THz emission properties in Fe/L1_0_-FePt/Pt trilayers compared to Fe/Pt bilayers, and we demonstrate that this enhancement is influenced by the formation of the L1_0_-FePt highly ordered phase at the magnetic/non-magnetic interfaces. To illustrate this, we grew extremely thin Fe (x)/L1_0_-FePt(2)/Pt(2) trilayers by means of ultra-high-vacuum co-sputtering technique. We achieved good control of the growth of the L1_0_ alloyed phase of specific thickness. We show that the presence of the L1_0_ phase between Fe and Pt layers drastically modifies the magnetic properties of the structures. Strong exchange coupling between soft magnetic Fe and hard magnetic L1_0_-FePt is observed, which becomes even stronger with increasing the Fe layer thickness. We show that the formation of an ordered L1_0_-FePt alloy phase promotes the interface transmission of spin current and amplifies the THz emission with respect to Fe(2)/Pt(3) bilayers. The enhanced spin-to-charge conversion in Fe/ L1_0_-FePt/Pt and the increased THz efficiency are crucial steps toward achieving emission performances similar to conventional THz sources.

## Figures and Tables

**Table 1:** Deposition parameters of the FM1/FM2/NM trilayer devices. Parameters of their fabrication as well as their nominal intended thickness are given.

Sample	Parameters	1st Layer Fe	2nd Layer FePt	3rd Layer Pt
S1	Power	50 W	Fe: 90 W/Pt: 30 W	40 W
	Temperature	300 °C	450 °C	450 °C
	Thickness	1.5 nm	2 nm	2 nm
	Working pressure (Ar)	5.8 × 10^−3^ torr	7 × 10^−3^ torr	7 × 10^−3^ torr
S2	Power	50 W	Fe: 85 W/Pt: 25 W	25 W
	Temperature	300 °C	450 °C	450 °C
	Thickness	4 nm	2 nm	2 nm
	Working pressure (Ar)	5.8 × 10^−3^ torr	7 × 10^−3^ torr	7 × 10^−3^ torr

## Data Availability

The data presented in this study are available on request from the corresponding author.
